# Content Analysis of Food Safety Information in Apple-Drying Recipes from YouTube, Blogs, Cookbooks, and Extension Materials

**DOI:** 10.3390/foods13050778

**Published:** 2024-03-01

**Authors:** Megan Low, Yaohua Feng

**Affiliations:** Department of Food Science, Purdue University, 745 Agriculture Mall Drive, West Lafayette, IN 47907, USA; low15@purdue.edu

**Keywords:** foodborne outbreaks, apple-handling practices, apple-drying recipes

## Abstract

Recurrent foodborne outbreaks associated with low-moisture foods prompted this study to evaluate apple-handling practices presented in apple-drying recipes available to United States consumers, and to explore the food safety implications of the recipes. Because little research is available on the safety of home fruit-drying, we conducted a systematic search of English-language apple-drying recipes from YouTube videos, blog articles, cookbooks, and university extension sources. Our evaluation found that most recipes excluded handwashing instructions, and potential cross-contamination practices were evident in 12% of the videos. Bruised or damaged apples were selected for drying in 16% of the videos, two blogs, and five cookbook recipes. Although more than half the blogs and videos demonstrated pre-treatment procedures, they did so predominantly to minimize browning with almost no mention of antimicrobial benefits. Drying temperature information was missing in 41% of the videos and 35% of the cookbooks that we evaluated. Even when temperatures were mentioned, most were insufficient for pathogen reduction according to the recommendations of previous studies. These videos, blogs, and cookbooks commonly advocated subjective indicators instead of unit measurements when slicing apples and checking for doneness. Our findings reveal the need for drastic improvements in food safety information dissemination to home apple-dryers and recipe developers.

## 1. Introduction

Many consumers prepare dried fruits in their home kitchens. Despite the perceived shelf-stability of dried fruits, an increase in foodborne illnesses from low-moisture foods (LMFs) worldwide has prompted a call for food safety information on preparing dried fruits at home. The CDC reported an outbreak of *Salmonella typhimurium* implicating dried coconut in 2017 [1], while dried apricot and freeze-dried apples were recalled by their manufacturers due to possible contamination with *Listeria monocytogenes* and *Salmonella*, respectively [2,3]. In South Africa and Norway, pathogenic bacteria, such as *Salmonella*, *Staphylococcus*, and *Clostridium*, have been detected in dried fruits [4,5].

The mishandling of foods by consumers in their home kitchens has been documented by many researchers [6,7,8,9,10]. A recent study also reported that consumers lack food safety awareness for apple-drying [11]. In addition to frequent consumer carelessness, such as inadequate handwashing and cross-contamination, unique food safety challenges are involved in the fruit-drying process. For example, fruit-drying utilizes low temperatures to minimize quality deterioration, but lower temperatures do not mitigate foodborne pathogens [12,13,14].

Blogs and social media platforms are popular avenues in which content creators share recipes. Those recipes also can be a source of food safety information for consumers [15]. Unfortunately, previous studies have shown that recipes have not been reliable sources of food safety information. Incorrect or vague instructions on handling procedures that could lead to cross-contamination have been found in video and blog recipes [16,17,18]. Food safety information from video and blog recipes also may be absent. Studies have documented missing information on endpoint temperatures and risk reduction behaviors in cookbooks when it comes to preparing meat and seafood [19] and missing guidelines in canning in food blogs [20]. Recipes for other low-moisture foods have been shown to be missing basic food safety practices, such as handwashing or using clean kitchen tools [17,18]. These recipes also excluded preventive measures specific to dried wood ear mushrooms and tree nuts, such as using boiling water during the soaking process to prevent microorganism growth [17,18].

Home fruit-dryers have access to an abundance of sources to obtain recipes, especially through internet searches. The Google search engine enables individuals to find information easily on any given topic of interest. In fact, Google was the most trafficked website in 2020 [21], followed by YouTube, a two-billion-user platform for video sharing [22]. The emergence of social internet platforms has enabled user-generated content. YouTube, for example, is an open-source platform in which anyone can create an account for free and upload their own videos. Blogs are another avenue in which users have the freedom to post self-written articles. Popular blogs are attracting up to five million readers a month. On the other hand, printed cookbooks are a more traditional avenue for recipe-sharing and have been a long-standing source for home cooks for a variety of reasons, including making familiar dishes or learning new skills [19,23]. Consumers who prepare food with wheat flour reported using cookbooks the most as their source of recipes in a survey [24]. Unsurprisingly, platforms that allow self-publishing can generate food safety information that is unregulated, and many cookbooks are authored by celebrity chefs who may not exemplify safe food-handling themselves [23]. In addition to these mainstream recipe sources, information about home fruit-drying is also available through U.S. Cooperative Extensions. To maintain their reach to the public, many university extension services have moved online, meaning home fruit-dryers can easily search for fruit-drying how-to information [25]. Unlike other sources, university extensions are handled by U.S. university faculty and disciplinary experts who strive to disseminate science-based information [26]. Little is known about the quality of food safety messages in fruit-drying recipes from university extensions. 

At the time this study was conducted, no existing evaluation of food safety information was available for dried-fruit recipes online or in physical cookbooks. This study assesses the food safety implications in fruit-drying recipes, using apple-drying as a case study, through a content analysis of videos, blogs, cookbooks, and university extension sources. Our objective was to gain insights into information that can promote safe fruit-drying practices and provide guidance to future food safety education development for home fruit-dryers.

## 2. Materials and Methods

### 2.1. Blog Selection

**Figure 1:**
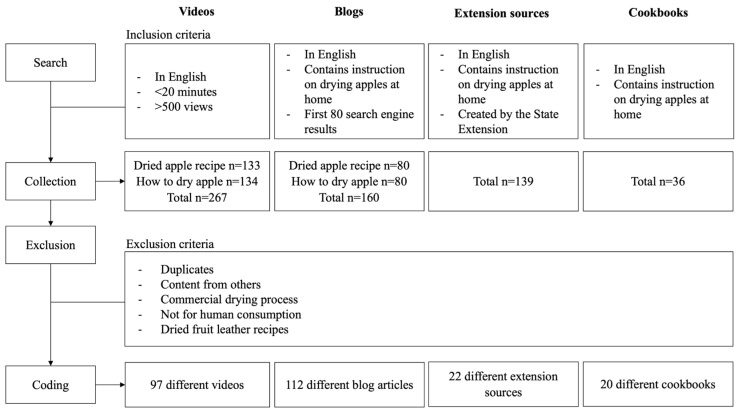
Selection process of each recipe source (videos, blogs, extension sources, and cookbooks) from collection to the final number of recipes included in the analysis.

We conducted a Google search in April 2021 using the keywords “how to dry apple” and “dried apple recipe”. Prior to the search, we compared different variations of the keywords using Google Trends in February 2021. We found that “how to dry apple” and “dried apple recipe” were the most popular search queries in Google among our tested keyword variations. We depersonalized the Google search by using the web browser’s “incognito” mode and turning off “signed-out search activity” in Google’s settings page. We collected the first 80 search results per keyword that met the following inclusion criteria and placed them into a Microsoft Excel spreadsheet: (1) content in English and (2) containing a home-based (non-commercial) recipe. This process resulted in a total of 160 blog articles from both keywords. We downloaded them as PDFs to preserve the information as of the collection date. Two researchers independently reviewed the downloaded blog articles to exclude recipes that met at least one of the following criteria: (1) duplicate recipes, (2) the use of another author’s recipe, (3) not containing instructions on drying apples at home, (4) dried apples not for human consumption (e.g., for craft), and (5) recipes for dried-fruit leather. A third researcher cross-checked individual exclusions to resolve any discrepancies. The selection process for all the materials is shown in Figure 1.

### 2.2. Video Selection

We conducted a YouTube search in February 2021 using the same keywords “homemade dried apple” and “dried apple recipe” for consistency. We sorted the search results for each keyword from highest to lowest view count to represent the most popular video recipes. We then collected videos that met the following inclusion criteria and entered them into the Excel spreadsheet: (1) content in English, (2) more than 500 view counts, and (3) less than 20 min. We screen-recorded these included videos to preserve the information as of the collection date. We applied the same exclusion protocol used for blog selection to the videos. 

### 2.3. Cookbook Selection

We conducted a cookbook search in June 2021 at local libraries within a two-mile radius of Purdue University, which included the Tippecanoe County Public Library and West Lafayette Public Library. The Purdue University Library housing recipe cookbooks was closed at the time of the search. At each library, two researchers manually browsed each book in the cookbook aisles for those that met the following inclusion criteria: (1) content in English and (2) cookbook title contains the word(s) “preservation”, “preserving”, “drying”, or “dehydration”. Researchers then checked for individual apple-drying recipes that contained a home-based (non-commercial) recipe by searching the index page using the words “apple”, “dehydrating”, “drying”, or “preserving”. If the words were not found on the index page, researchers checked the table of contents for sections related to drying or dehydrating fruit and manually browsed the pages in these sections for home-based apple-drying recipes. If they did not find any home-based apple-drying recipes, they excluded the cookbook. They also applied the other exclusion criteria used for blog selection to the cookbooks. Researchers captured photos of the pages in the included cookbooks and stored them for future reference.

### 2.4. University Extension Source Selection

We identified university extension websites through the United States Department of Agriculture’s (USDA’s) National Institute of Food and Agriculture website [27]. As information is typically organized differently on each website, we employed three search methods to ensure a thorough search. We adapted methods from Boehm’s (2015) [28] content analysis of food traceability information in U.S. extension websites. First, we conducted a manual search using the navigation links on the homepage to locate webpages containing home apple-drying procedures. The links were usually “home food preservation” or “drying fruits at home”. Our second method involved using the website’s search field, if one was available on the homepage. We entered keywords “drying fruit” and “dehydrating fruit” into the search field separately. Unlike the blogs and videos where we aimed for the most popular recipes, the goal of this search was to exhaustively browse the apple-drying content from each university extension website. Therefore, we chose broader keywords to prevent filtering out potential content. Lastly, if a “publications” page was available on the homepage, the researcher navigated to the page and conducted another search within the publication directory using the same keywords. If any of the search methods did not produce recipes that met the inclusion criteria, the researcher indicated this on the spreadsheet. We showed the search method used for all the extension sources collected. We downloaded the extension sources as PDFs to preserve the information as of the collection date. Finally, we used the same exclusion protocol that we used for blog selection and applied it to the extension sources. 

### 2.5. Further Exclusion Process

We collected a total of 160 blog articles, 267 videos, 36 cookbooks, and 139 extension sources during the search. After excluding duplicates between keyword and library searches, we removed 40 blogs, 61 videos, 1 cookbook, and 41 extension sources. We also excluded 5 blogs, 108 videos, 14 cookbooks, and 28 extension sources because they did not provide instructions on how to dry apples (e.g., baked goods recipe with dried apple as an ingredient). Other exclusion criteria led to the removal of three blogs because the dried apples were for crafts instead of human consumption. We removed one video because the instructions were not in English, one cookbook because the apples were processed with an additional heat step to make dried-apple leather, and one extension source because the PDF file could not be opened. We also removed 47 extension sources that were not publications, like blog articles, slideshow presentations, and videos.

### 2.6. Coding System

We adapted a coding system from previous content-analysis literature [21,29] and systematically collected, coded, and placed information into a Microsoft Excel spreadsheet. The three main categories of the coding system were: (1) source information, (2) user interactions (for blogs and videos), and (3) apple-handling practices and rationale for the practices. Using our source information, we characterized recipes by their title, author, published date, and location. User interactions differed depending on the online platform. For blogs, we recorded average recipe ratings, number of shares, and number of comments. For videos, we recorded the number of YouTube channel subscribers, number of video views, number of likes, and number of comments. User interactions were not available from printed cookbooks and university extension websites. Our researchers used the Site Explorer feature on Ahrefs SEO Tool (Santa Barbara, CA, USA, 2021) to obtain average monthly website visits for blogs and extension websites. We developed individual coding items in the apple-handling practices category based on the steps found in the blog and university extension sources of a preliminary search. These included: (1) selection criteria of fresh apples, (2) handwashing, (3) apple-washing, (4) peeling, slicing, and coring apples, (5) apple pre-treatment, (6) apple-drying, (7) doneness check, (8) dried-apple storage, and (9) usage of dried apples. To prevent bias, two trained researchers independently coded the information into locally downloaded versions of the Excel spreadsheet. A third trained researcher reviewed the two sets of coded information. If there were any discrepancies between the two sets of information, the third researcher resolved them by checking the source directly or brought the discussion to the main researcher for further consensus. We qualitatively coded all information to prevent losing context in numerical forms, but we later unitized it for quantitative analysis [30,31]. Because this study is exploratory, the main researcher unitized the coded qualitative data inductively. For example, the apple selection criterion “great way to use up spotted or bruised apples” was assigned “1” under the variable “bruised”. If there were any subjective interpretations, a second researcher confirmed the variable assignment.

### 2.7. Data Analysis

We employed both quantitative and qualitative analyses and adapted the data analysis procedure from previous studies [16,21,31]. Our team conducted descriptive analysis in Microsoft Excel 2011 (Microsoft Corporation, Redmond, WA, USA) to quantify the frequencies of practices or rationale for practices coded in the recipes, and summed and converted the coded items to percentages. 

## 3. Results and Discussion

The study’s search and exclusion processes resulted in 112 blog articles, 97 videos, 20 cookbooks, and 22 extension sources that were included in the analysis (Figure 1). We found that “How to Make Dried Fruit at Home (Using Your Oven) by Natural Cures” was the video with the most views [32], and “Homemade Cinnamon Apple Chips” by Carrie’s Experimental Kitchen was the blog article with the most website visits [33].

### 3.1. Pre-Drying Steps

**Figure 2:**
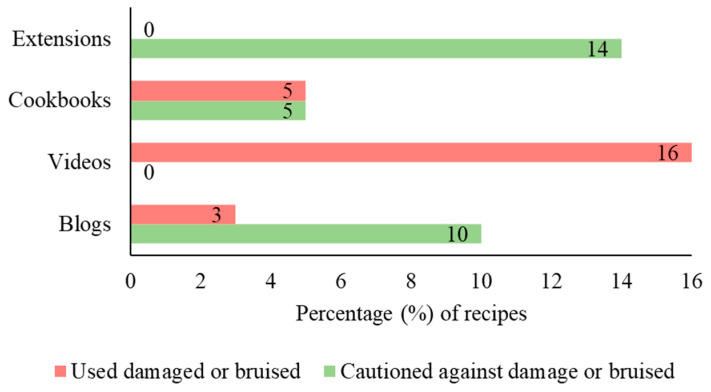
Proportion of recipes within the four content sources that used damaged or bruised apples (red) and those that cautioned against damage or bruised apples (green).

Recommendations about how to select or source apples for drying were missing from most recipes (videos, 70%; blogs, 43%; cookbooks, 30%; extension sources, 27%). Very few recipes (extension sources, 14%; blogs, 10%; cookbooks, 5%; videos, 0%) cautioned against using bruised or damaged apples (Figure 2). Extension sources had the most mentions on avoiding bruised apples (Figure 2). On the contrary, some videos (16%) and blogs (3%) suggested using bruised or damaged apples. When we assessed the recipe authors’ rationales for selecting bruised apples, two of the blogs suggested drying apples as a “great way to use up” bruised, mealy, or spotted apples and another two recommended using apples that were “seconds”. One video creator mentioned bruising was acceptable because the apples were being cooked, while a cookbook said it was acceptable “as long as the bad parts were cut out”. Two videos mentioned obtaining “imperfect apples for free” or that imperfect apples were “cheaper due to bruising”. These reasons provided the consumers with motivation for using bruised apples for drying, which is not advised based on microbial validation studies that found the following evidence: (1) bruised apples or fruits can be contaminated by foodborne pathogens; (2) by cutting out the bruised or damaged parts, the internalized pathogens cannot be removed [34]; and (3) without reaching a valid temperature, desiccation is not lethal to pathogens [35,36].

Washing apples before drying can remove soil and pathogens on the surface. Rinsing apples under tap water has been found to be effective in removing 1 to 3 logs of foodborne bacteria, fungi, and viruses [37,38]. However, almost half the blogs (48%), three-quarters of the videos, over a third (35%) of the cookbooks, and 18% of the extension sources skipped this step. Most of the recipes that included washing the apples did not describe the steps on how to properly wash them; instead, they only mentioned “wash the apples” or “use a clean apple” (Table 1). Some of the blogs, videos, and cookbooks that described a wash method recommended using soap, vinegar, or baking soda (Table 1). This is consistent with a previous consumer survey and content-analysis findings that indicate the public is unaware of the proper way to wash fresh produce, often using unrecommended methods like soap [21,39,40]. Soap is effective in removing bacteria in handwashing, but ingesting residual soap on the fruit can make consumers sick [41].

Apples are often sliced to prepare them for drying, but we found that measurements on apple slice thickness were missing in many recipes, with videos (79%) lacking this information more than other sources (Table 1). Some video (32%) and blog (20%) recipes without unit measurements provided vague descriptors of the thinness of slices, like “wafer thin”, “paper thin”, or “pseudo thin”. Researchers observed five videos without measurements that showed slicing the apples thickly. For example, an apple was divided into about four rings. In two other videos, apples were sliced into wedges. Previous studies showed that the thickness is an important parameter that determines the drying rate [42,43]. The thicker the apple slices, the longer it takes for moisture to migrate from the center to the surface for evaporation [35,42]. At the time of this study, no published microbial validation studies had been conducted to provide guidance to home apple-dryers on the combinations of slice thickness, drying time, and temperature.

Most cookbooks (90%) and extension sources (95%) presented a pre-treatment step, but many blogs (32%) and videos (36%) were lacking that step. Of those that provided pre-treatment instructions, some proposed the step as optional (cookbooks, 22%; blogs, 13%; videos, 3%). However, none of the extension sources classified pre-treatment as optional. Immersing apple slices in an acidic solution was the most common pre-treatment method (Table 2). Lemon juice was suggested most in blogs, videos, and cookbooks, and ascorbic acid was suggested the most in extension sources, but many recipes showed this step as optional. Pre-treatment was predominantly mentioned in recipes to minimize the browning effect, with comments like “I am not concerned with colour change” or “a little browning shouldn’t affect the flavour”, implying that recipe authors assumed that pre-treatment was only for aesthetic enhancement. Only a few sources (extension sources, 6; cookbooks, 3; blogs, 4; videos, 0) mentioned the potential food safety benefits (Table 2). It is important for recipes to emphasize the antimicrobial benefits. A survey found that microbial control was the most important reason for home apple-dryers who did not pre-treat to consider adopting the practice [11]. Growing evidence indicates pre-treatment as a good practice to mitigate foodborne pathogens. Research shows that apple dehydration following pre-treatment with acids or sulfites achieved more than five log reductions in *Salmonella* and *Escherichia coli* O157:H7 [14,44,45]. With heated ascorbic acid, immersion for just 90 s was sufficient to produce a 7.9 log reduction in *Salmonella* [46]. While there are no published data on apple dehydration in particular, steam and hot water blanching of other produce for three minutes showed an equivalent log reduction effectiveness in *Salmonella* and *L. monocytogenes* [47]. Additionally, hot water or hot acetic acid immersion for one to three minutes was shown to reduce rot by *Penicillum expansum* on fresh apples [48].

### 3.2. Apple-Drying Process

Whether the apple slices are spread out evenly or overlapping on top of one another could affect the drying rate and uniformity, but very few recipes described how to arrange them on the drying tray. A high proportion of blogs (79%), videos (94%), cookbooks (80%), and extension sources (82%) did not mention avoiding an overlap of slices. One video creator suggested that there is “no set method” to arrange the apple slices but indicated a preference for overlapping apples. Only five blogs, two videos, and two cookbooks explained the importance of avoiding overlap for air circulation or drying uniformity.

**Figure 3:**
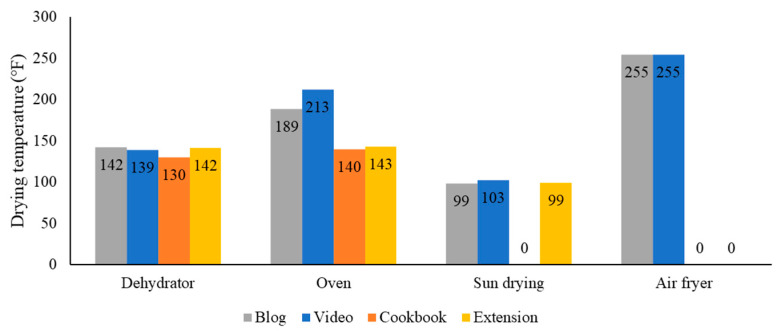
Average drying temperatures (°F) recommended by blogs (gray), videos (blue), cookbooks (orange), and extension sources (yellow) that provided a temperature.

**Figure 4:**
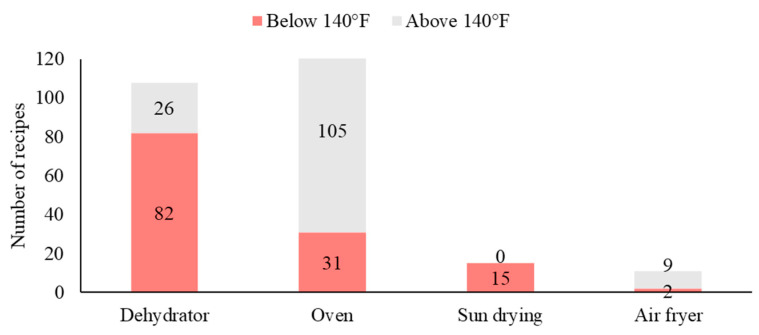
Number of recipes from blogs, videos, cookbooks, and extension sources that recommended drying temperatures above 140 °F (gray) and below 140 °F (red).

To dry the apples, dehydrators and ovens were most often used or recommended in recipes (extension sources, 86% and 82%; cookbooks, 70% and 40%; blogs, 54% and 88%; videos, 46% and 32%, respectively). Sun-drying was the next most common method (Table 3). Drying temperatures were missing in many recipes (videos, 41%; cookbooks, 35%; extension sources, 9%; blogs, 8%). When temperatures were provided, the average drying temperatures varied by drying method and recipe sources (Figure 3), with a majority of recipes recommending temperatures below 140 °F (60 °C) (Figure 4). Some previous studies indicated that 140 °F was not effective in killing pathogenic bacteria during apple-drying [35,49]. Grasso-Kelley et al. (2020) [49] found that drying at 219 °F (104 °C) and 275 °F (135 °C) resulted in more than five log reductions in *Salmonella* in dried apples. However, it can be challenging for home-use dehydrators to achieve these high temperatures. Observations from videos showed that home-use dehydrators are often built with either pre-set or even no controls over drying parameters. One of the dehydrators in the videos had only an on-and-off switch, and another dehydrator had a “fruits and vegetables” setting pre-set at 135 °F (57.2 °C).

These challenges were similar for sun-drying. It was common to sun-dry in the open air by laying apples on a flat surface or hanging them on a string (67% of sun-drying recipes). Only about a fifth of sun-drying recipes suggested using solar dryers. When sun-drying apples in the open air, apples are subject to ambient temperatures that typically do not go above 109 °F (43 °C), based on previous experiments [50,51,52]. During open-air sun-drying, the surface temperature of fruits can increase beyond the ambient temperature but only by a few degrees [50]. The use of a solar dryer can considerably raise the temperature within the dryer, up to temperatures of 177 °F (80.5 °C) [53,54]. However, access to special equipment can be a barrier for home apple-dryers.

It is important to balance the drying temperature with drying time for microbial control. In recipes that recommended temperatures 140°F and below, the most common drying time was 12 h for the dehydrator (37%), 6 h for the oven (11%), and 48 h for sun-drying (22%). We were concerned by recipes suggesting a drying time as short as 2 h in the dehydrator and 45 min in the oven at these low temperatures. A laboratory study reported that 4.5 h of convective drying at 140 °F (60 °C) was needed for apple slices to achieve a water activity below 0.4 [55]. At 122 °F (50 °C), Royen et al. (2020) [56] reported the shortest drying time was 4.7 h at their highest air velocity to achieve a similar water activity. At a lower air velocity, it took 9 h. Drying at home without convection or with thicker apple slices than the experiments will require an even longer drying time. Overall, the drying time recommendations in the recipes varied widely, as much as five days between recipes and 24 h within the same recipe. This introduces an additional challenge for home apple-dryers in determining a specific time to follow.

We found that the relative humidity of the drying environment influences drying rates and pathogen thermal inactivation; however, the recipes providing information on humidity were scarce. Only 22% of the sun-drying recipes provided a humidity level of 60%, and one recipe suggested a humidity level of 20%. A humidity level of 60% was recommended by a drying methods review, but the paper stated that its focus was on drying efficiency, quality preservation, and cost-effectiveness [57]. From a food safety perspective, a low humidity will lengthen the time needed to achieve pathogen log reductions, while a high humidity can produce greater microbial inactivation, even with a lower internal product temperature [58,59]. A study on high-humidity apple-drying reported a sharp increase in *Salmonella* log reduction once the relative humidity in the heating chamber had built up [46]. That study achieved effective *Salmonella* log reduction with a relative humidity of 62% at 158 °F (70 °C) and 74% at 194 °F (90 °C).

Most of the recipes did not specify how to determine the doneness of the apples. Among the recipes that provided a doneness description, inspection by touch was the most common across all sources. Some descriptive terms that were used to imply the doneness were “leathery”, “crisp”, or “pliable” (Table 3). Other doneness descriptions included “easy to snap in half” and visual indicators, such as “looking” for moisture, ruffled edges, shrinking, or color. Only one extension source provided an endpoint moisture content of 20% for dried apples but did not include how to measure it. There is a lack of validation data to support moisture content levels and the subjective indicators. One study reported that after reaching a water activity below 0.85, dried apples still had not attained the recommended five log reduction in *Salmonella* for safe consumption [49]. In the present study, no recipe mentioned using water activity as a determining indicator for doneness.

The researchers were unclear about how to choose which parameters to use to determine the doneness of drying to ensure food safety. As a moisture-content or water-activity meter is not accessible equipment to most home apple-dryers, it may seem more practical for home apple-dryers to use tactile, taste, and visual indicators to determine doneness. However, those indicators can be very subjective, and the interpretation can vary from person to person. Misinterpretation can lead to apple slices that are too thick to sufficiently dry out to a low water activity that prevents microbial growth. Levine et al. (2017) [23] uncovered similar findings in cookbook recipes for egg doneness, stressing that subjective indicators often lead to incorrect judgment.

### 3.3. Storage and Usage of Dried Apples

Instructions on cooling before storage were not presented in many recipes (cookbooks, 91%; videos, 80%; blogs, 44%; extension sources, 32%). One blog instructed to package the dried apples “immediately”, implying no cooling step. The cooling step is important, because storing dried apples immediately can lead to condensation formation in the packaging and promote the growth of microorganisms [60,61].

The proposed storage duration varied vastly among recipes, from three days to an extensive timeframe, such as “25 years”, “years and years”, or “forever, if stored tightly”. While dried apples are relatively shelf-stable, an exaggerated duration is misleading because storage conditions (e.g., temperature, relative humidity) and packaging influence the duration that allows for safe consumption [62,63]. As for the conditions for storage, most recipes recommended a “cool, dark, or dry place” (Table 4), and only two cookbooks and one extension source specified storage temperatures of 50 to 70 °F (10–21 °C). This allows subjectivity in how home apple-dryers interpret their method of storage. In fact, a survey showed that almost 90% of home apple-dryers in the U.S. did not monitor their storage humidity and temperature [11]. Another survey on tree nuts showed evidence that consumers varied widely in their storage temperatures and storage lengths of tree nuts, some of which could support the survival of pathogens [64]. Home apple-dryers can face the same issue in using unsafe practices without science-based recommendations.

For a low-moisture food like dried apples, packaging provides an important barrier to moisture absorption. Jars and plastic bags were common recommendations, but not all recipes specified whether they were to be airtight, vacuum-sealed, or freezer-weight (bags) (Table 4). Freezer-weight bags were more common in extension sources (36%) than blogs (3%), videos (1%), and cookbooks (5%). A few videos (4%) even suggested storing dried apples in an open bowl or container without any seal. Previous studies have showed that airtight and sealed packaging can reduce the moisture within the packaging and slow possible microbial growth [62,63]. Extension sources promoted freezer-weight bags, which are made of thicker plastic, for their moisture-proof quality [65]. There is a lack of data on dried-apple storage conditions and packaging; however, a previous study on dried pumpkin shows storage in open bags produced twice the amount of yeast and mold than storage in closed bags [63].

Blogs and videos mostly suggested the consumption of dried apples that does not involve a heating step, including eating directly, as a topping on cereal, or adding to a dry mix like granola (Table 4). One cookbook suggested rehydrating the dried apples in a cold-water soak overnight, which can provide an adequate environment and time for dormant microbial cells to reactivate and grow [66]. The final use of dried apples without a further heating step only underscores the need for safe food practices throughout the process from start to finish. 

### 3.4. Handwashing and Cross-Contamination

Handwashing instructions were rarely presented in the recipes (blogs, 3; extension sources, 2; videos, 0; cookbooks, 0). Two blogs that mentioned handwashing did not provide details of instructions, they only noted “always wash your hands before preparing food” and “just use clean hands”. It was alarming that handwashing was omitted in the recipes, despite its effectiveness in preventing the transfer of bacteria [67,68]. Unfortunately, this has been found to be a common occurrence in other recipes as well. Handwashing was missing from all blog and video recipes on wheat flour and eggs, and 99% of video recipes on dried wood ear mushrooms [16,17]. Although handwashing is an everyday practice, studies have unveiled that there are food preparers who do not know that scrubbing for 20 s and using hot water is the proper handwashing technique [69,70]. Incorporating handwashing instructions can be helpful for food handlers using the recipes. A study showed that participants who received food safety instructions within their recipe had improved safe-handling practices [71]. Resources have been developed to promote the inclusion of food safety instructions. The Partnership for Food Safety Education has created the Safe Recipe Style Guide for recipe authors to use [72]. 

Cross-contamination events were observed in 12% of the videos, which included preparing apples directly on unsanitized counters, putting non-food-contact objects on the food-contact surface, touching non-food-contact surfaces and continuing working, and drying raw meat in the same device at the same time as the apples without sanitization. Similarly, cross-contamination events have been reported in video recipes preparing other foods [73,74,75]. Cooking competition shows evaluated in Borda et al.’s (2014) [74] study captured contestants’ continuous movement in the kitchen, which allowed the observation of chefs tasting the dish along the way or wiping their hands on their towel or apron. YouTube videos, which are the videos that were analyzed in the present study, are usually short clips of longer footage and may leave these behaviors out. Even so, visual representation allowed the researchers to identify cross-contamination events that would otherwise be missed in blogs, cookbooks, and extension sources, whose instructions were described in text.

### 3.5. Food Safety Information in Different Recipe Sources

Blogs, videos, cookbooks, and extension sources displayed different food safety information accuracy, including writing style throughout the recipe. Blog and video recipes covered less food safety information and had an unconcerned tone regarding food safety aspects, compared to cookbooks and extension sources. Some cookbooks included sections or whole chapters about food safety, pre-treatment, or drying methods [76,77,78]. It is common for social media platforms, like blogs and YouTube, to include inaccurate scientific information. This is prevalent throughout many science topics, including food safety [21,40,79] and others like environment and health [80,81,82].

The difference in food safety information could stem from authors’ varying backgrounds, some of which may not involve food safety training. Open-access platforms like blogs and YouTube allow anyone to create and share content, including amateur recipe authors who are not experts in the drying process themselves. One video creator mentioned they “did not know for years” that there was a temperature dial on their dehydrator. When using the microwave, another video creator said, “I don’t know what [microwave power] means, I really don’t care”. Although many of the authors of the information sources in the present study did not explicitly state their profession, similar studies evaluating jam and flour recipes confirmed that food bloggers and video creators are predominantly non-professionals [16,83]. Brodt (2020) [83] highlighted that more amateur creators provided less food safety information in YouTube videos.

The difference in food safety information could also result from the different objectives of each platform. Some food bloggers describe blogging for leisure, like self-documentation of the foods they cook or eat, while others use it to form a community or a source of income [84,85,86]. The trend of building a following and content commercialization has also been shown to be an increasingly influential motivation for video content creation [87]. Hence, the creators of blogs and videos would be interested in attracting readers or viewership. In the present study, the cookbooks were topic-focused and served as specialized information handbooks. Meanwhile, the objective of commercialization does not apply to extension services whose primary goal is to educate the public on food safety. For these reasons, cookbooks and extension sources may be more directive and include more food safety information.

### 3.6. Limitations

Even with careful design and execution, this study encountered limitations. A singular metric to define the popularity of online sources was difficult to establish [88]. In the present study, we assumed view count for videos and top Google search results for blogs as proxies for this. While average webpage visits from Ahrefs SEO Tool were additionally collected, Ahrefs states that traffic numbers are estimates and vary among different SEO services. Furthermore, these average website visits accounted for the whole website domain and not the recipe page itself. User interactions were not always available on blogs, depending on how the page was designed. This resulted in a limited sample size when conducting statistical analyses. Lastly, cookbooks evaluated in the present study only encapsulate the researchers’ location due to physical travel limitations. The selection of cookbooks in libraries will vary in different locations; hence, our findings cannot be generalized to all cookbooks.

## 4. Conclusions

In summary, many recipes lack food safety communications. The motivators of the instructions are better taste or texture rather than food safety. The food safety instructions from apple-drying recipes were neither consistent nor accurate. Home apple-dryers can be confused as to what best practices they should follow. Food safety risks are unique to apple-drying in a domestic kitchen setting and call for the development of tailored consumer food safety education for recipe developers and home apple-dryers alike. The scarcity of microbial validation studies examining the conditions of the current apple-drying processes makes it challenging to provide evidence-based recommendations at this time. More scientific validation of apple-drying procedures can help recipe developers standardize their recommendations.

## Figures and Tables

**Table 1:** Measurement of apple slice thickness given by recipes in blogs, videos, cookbooks, and extension sources.

	Blogs (n = 112)		Videos (n = 97)		Cookbooks (n= 20)		Extensions (n = 22)	
	%	(n)	%	(n)	%	(n)	%	(n)
Apple-washing method								
No mention	48	(54)	75	(73)	35	(7)	18	(4)
“Wash” or “clean” without method	38	(42)	7	(7)	60	(12)	68	(15)
Rinse under running water	5	(6)	15	(15)	5	(1)	14	(3)
Soap and water	2	(2)	1	(1)	10	(2)	0	(0)
Homemade soak	1	(1)	1	(1)	0	(0)	0	(0)
Vinegar rub	1	(1)	0	(0)	0	(0)	0	(0)
Soak in water	0	(0)	3	(3)	5	(1)	0	(0)
Apple slice thickness								
No measurement given	28	(31)	79	(77)	20	(4)	18	(4)
Less than ¼ inch	32	(36)	10	(10)	20	(4)	50	(11)
¼ inch–½ inch	39	(44)	10	(10)	65	(13)	55	(12)
More than ½ inch	2	(2)	0	(0)	0	(0)	0	(0)

**Table 2:** Pre-treatment methods for apple slices from recipes in blogs, videos, cookbooks, and extension sources.

	Blogs (n = 112)		Videos (n = 97)		Cookbooks (n = 20)		Extensions (n = 22)	
	%	(n)	%	(n)	%	(n)	%	(n)
Pre-treatment method								
Acidic solution	64	(72)	45	(44)	75	(15)	82	(27)
Lemon juice	61	(68)	41	(40)	55	(11)	27	(6)
Citric acid	13	(15)	10	(10)	15	(3)	27	(6)
Ascorbic acid	11	(12)	2	(2)	45	(9)	82	(18)
Other fruit juice	4	(5)	2	(2)	30	(6)	5	(1)
Pre-dry with kitchen or paper towel	24	(27)	11	(11)	10	(2)	5	(1)
Salt water	6	(7)	9	(9)	0	(0)	18	(4)
Vinegar	5	(6)	6	(6)	0	(0)	0	(0)
Syrup or honey dip	5	(5)	2	(2)	10	(2)	27	(6)
Blanch in syrup or water	5	(5)	5	(5)	10	(2)	32	(7)
Sulfite solution	2	(2)	1	(1)	10	(2)	55	(12)
Reason for pre-treatment								
Anti-browning	54	(61)	37	(36)	75	(15)	91	(20)
Flavor	10	(11)	3	(3)	10	(2)	14	(3)
Texture	5	(6)	0	(0)	0	(0)	5	(1)
Reduce moisture	4	(5)	2	(2)	5	(1)	0	(0)
Antimicrobial	4	(4)	0	(0)	15	(3)	27	(6)
Nutrition retention	4	(4)	0	(0)	5	(1)	18	(4)

**Table 3:** Drying method and doneness check recommended from recipes in blogs, videos, cookbooks, and extension sources.

	Blogs (n = 112)		Videos (n = 97)		Cookbooks (n = 20)		Extensions (n = 22)	
	%	(n)	%	(n)	%	(n)	%	(n)
Drying method								
Oven	79	(88)	32	(31)	40	(8)	82	(18)
Dehydrator	54	(60)	46	(46)	70	(14)	86	(19)
Sun-drying	13	(15)	11	(11)	35	(7)	59	(13)
Indoor air drying	10	(11)	14	(14)	10	(2)	9	(2)
Microwave	4	(4)	2	(2)	0	(0)	0	(0)
Air fryer	3	(3)	4	(4)	0	(0)	0	(0)
Freeze dryer	0	(0)	7	(7)	0	(0)	5	(1)
Pan	0	(0)	2	(2)	0	(0)	0	(0)
Freezer	0	(0)	1	(1)	0	(0)	0	(0)
Doneness check								
Textural endpoint	71	(79)	23	(22)	90	(18)	86	(19)
Dryness endpoint	38	(42)	5	(5)	35	(7)	68	(15)
Color endpoint	3	(3)	3	(3)	0	(0)	5	(1)
No method	63	(70)	85	(82)	75	(15)	55	(12)
Touch inspection method	30	(34)	7	(7)	15	(3)	46	(10)
Visual inspection method	11	(12)	2	(2)	5	(1)	23	(5)
Bend or snap method	6	(7)	7	(7)	15	(3)	18	(4)
Taste method	2	(2)	1	(1)	5	(1)	0	(0)

**Table 4:** Storage and usage of dried apples.

	Blogs (n = 112)		Videos (n = 97)		Cookbooks (n = 20)		Extensions (n = 22)	
	%	(n)	%	(n)	%	(n)	%	(n)
Storage location								
Cool, dry, dark place	15	(17)	5	(5)	20	(4)	82	(18)
Freezer	13	(15)	8	(8)	5	(1)	23	(5)
Refrigerator	4	(5)	4	(4)	0	(0)	14	(3)
Room temperature	4	(4)	9	(9)	10	(2)	9	(2)
Packaging type								
Airtight packaging	41	(46)	12	(12)	35	(7)	14	(3)
Jar	20	(22)	23	(22)	15	(3)	68	(15)
Plastic bag	11	(12)	22	(21)	15	(3)	64	(14)
Vacuum-sealed packaging	8	(9)	6	(6)	10	(2)	9	(2)
Freezer-weight bags	3	(3)	1	(1)	5	(1)	36	(8)
Open bowl/container	0	(0)	4	(4)	0	(0)	0	(0)
Dried-apple usage								
Without heat step	57	(64)	38	(37)	55	(11)	45	(10)
On its own	54	(61)	35	(34)	50	(10)	36	(8)
As a topping (e.g., on cereal, oatmeal)	17	(19)	8	(8)	10	(2)	18	(4)
In a dry mix (e.g., granola or trail mix)	16	(18)	4	(4)	20	(4)	14	(3)
In cold drinks	1	(1)	1	(1)	0	(0)	5	(1)
With heat step	16	(18)	15	(15)	60	(12)	59	(13)
Baked or cooked recipe	15	(17)	11	(11)	50	(10)	36	(8)
Rehydrated	5	(6)	7	(7)	30	(6)	50	(11)
In hot drinks	3	(3)	3	(3)	0	(0)	5	(1)

## Data Availability

The original contributions presented in the study are included in the article, further inquiries can be directed to the corresponding author.
